# Effects of subjective successful aging on emotional and coping responses to the COVID-19 pandemic

**DOI:** 10.1186/s12877-021-02076-2

**Published:** 2021-02-17

**Authors:** Dannii Y. Yeung, Edwin K. H. Chung, Alfred H. K. Lam, Alvin K. K. Ho

**Affiliations:** Department of Social and Behavioural Sciences, City University of Hong Kong, Kowloon, Hong Kong

**Keywords:** Coronavirus disease, Subjective perceptions of successful aging, Perceived time limitation, Emotional and coping responses

## Abstract

**Background:**

Middle-aged and older adults are more vulnerable to hospitalization and mortality if they are infected with the COVID-19 virus. The present study investigates the longitudinal effects of subjective successful aging on middle-aged and older adults’ emotional and coping responses to the COVID-19 pandemic, and explores an underlying mechanism through perceived time limitation during the pandemic.

**Methods:**

A sample of 311 Hong Kong Chinese middle-aged and older adults (*M*_*age*_ = 64.58, *SD* = 10.14, Range = 45–90 years) were recruited from an Adult Development and Aging Project and participated in a questionnaire study via an online platform or phone interview. Their levels of subjective successful aging, perceived time limitation, and emotional and coping responses to the pandemic were measured.

**Results:**

The respondents who perceived themselves as more successful in aging process reported more positive and fewer negative emotions compared with their counterparts with lower levels of subjective successful aging. The mediation analysis showed that perceived time limitation could partially account for the effects of subjective successful aging on emotional and coping responses.

**Conclusions:**

Findings of this study unveil the beneficial effects of subjective views of successful aging on emotional and coping responses to the pandemic through alleviating their perception of time limitation.

**Supplementary Information:**

The online version contains supplementary material available at 10.1186/s12877-021-02076-2.

## Background

A novel infectious disease named the coronavirus disease 2019 (COVID-19) was first identified in December 2019 in Wuhan, China, and has spread globally from February 2020 onwards. The World Health Organization (WHO) formally announced COVID-19 as a pandemic on 11 March 2020. In response to this pandemic, multiple preventive measures have been implemented in Hong Kong (HK) since late January 2020. For example, “work from home” arrangements were implemented for civil servants between 29 January and 3 May 2020. Similar measures have also been adopted in private and public organizations. Moreover, social distancing measures have been implemented since 28 March 2020, including the temporary closure of public entertainment places, such as theatres and fitness centers [1]. High perceived uncertainty and susceptibility among local residents were likewise observed because of the lack of information on the means of infection and effective treatment in the early stages of the pandemic [2]. The acute surgical mask shortage at the onset of the outbreak also precipitated panic and anger, and social distancing measures considerably interrupted work arrangements and social relationships. As of 11 March 2020, there were 129 confirmed cases in HK, with a mean age of 55.9 (*SD* = 16.9; Range = 16–96) [3], thereby suggesting that middle-aged and older adults are the most vulnerable age groups to this disease. Therefore, the present study aims to identify the factors influencing their emotional and coping responses to the COVID-19 pandemic.

### Successful aging

The literature on successful aging has provided a fundamental theoretical basis to understand middle-aged and older adults’ responses to the unexpected and life-threatening COVID-19 pandemic. Despite the lack of consensus on a formal definition for successful aging, researchers generally agree that successful aging appears as a multicomponent concept that often includes relatively good physical and mental functioning [4–6]. Rowe and Kahn’s three major objective criteria [7] have been widely adopted among the many conceptualizations for successful aging: the absence of disease and disease-related disability, maintenance of high cognitive and physical functioning, and active engagement with life. Their definition of successful aging has facilitated the differentiation of successful agers (i.e., older adults whose health and social adjustments are above average) from normal agers, and inspired numerous studies from diverse disciplines to examine the prevalence and correlates of successful aging globally [8, 9]. Although this conceptualization of successful aging is popular, it has been criticized for its biomedical focus and lack of lay perception in defining what constitutes successful aging [10, 11].

Given these criticisms on Rowe and Kahn’s conceptualization for successful aging, more attention has been redirected to the field of subjective successful aging [12–14]. Aging is a dynamic lifelong process, such that an individual may perceive themselves having aged successfully at one point in their lives but not at others [15], or being successful in one domain of successful aging but not the others [16]. Subjective successful aging is considered as a self-appraisal of their own aging process over multiple dimensions of later life [13, 17]. The self-evaluation procedure of successful aging involves simultaneous weighting over multiple aspects of later life as well as comparing their own aging trajectory with their peers [18], therefore, it is more comprehensive and precise in reflecting older individuals’ perception of their own aging process. While further investigations for the conceptualizations of successful aging among laypeople are needed, in the current state, studies often utilize a single item to measure subjective successful aging [17, 19, 20]. Evidence has suggested that self-rated successful aging had stronger associations with older adults’ well-being than Rowe and Kahn’s objective criteria of successful aging [19–21]. Studies also consistently reveal that higher levels of self-rated successful aging are associated with better physical functioning, higher levels of resilience, and fewer depressive symptoms [17, 19]. In a longitudinal study conducted over the course of 23 years, positive self-perception of aging was found to contribute to the survival rate of Americans [22].

Previous studies have demonstrated that a positive view of aging is one of the major protective factors against the negative effects of aging on physical and psychosocial well-being. For example, Dutt et al. showed that the awareness of age-related gains and losses are linked to the use of adaptive regulatory behaviors such as assimilation and accommodation [23]. Using a hypothetical risk-taking task, Brassen et al. demonstrated that successful agers exhibited a greater tendency to regulate emotions than their non-successful counterparts [24]. Moreover, relative to normal agers, successful agers have been found to actively utilize adaptive strategies (such as loss-based selection) to manage their negative emotional experiences [25] and proactively deal with future stressors to prevent the occurrence of negative events or potential losses [26, 27]. This reveals a potential linkage between subjective successful aging and the use of problem-focused coping. Inferring from these findings, it is therefore anticipated that individuals with a higher self-rating of successful aging may prefer problem-focused coping over emotion-focused coping, and may exhibit improved affective well-being in response to the pandemic.

Evidently, people of older age, particularly those with chronic diseases, are significantly vulnerable to the COVID-19 pandemic and its impacts. The implementation of social distancing measures further restricts social activities of older people, creating a potential threat to their social and psychological well-being. The COVID-19 pandemic thus provides a platform to examine the long-term impacts of subjective successful aging on response patterns in the face of a negative natural event, and to investigate whether perceived time limitation caused by the pandemic can be one of the underlying mechanisms. Accordingly, the present study investigates whether middle-aged and older adults with higher scores of self-rated successful aging would exhibit better adjustment during the pandemic.

### Perceived time limitation

Previous research on the responses facing unexpected and uncontrollable crises such as September 11 attacks in the United States and the SARS outbreak in Hong Kong has provided a salient reminder that life is finite [28]. The same situation should be applicable to the current COVID-19 pandemic. Despite the lack of studies directly linking subjective successful aging and perceived time limitation together, individuals’ views of their own aging process provide them with a frame for future expectations and their perceived controllability in the face of certain events, subsequently influencing their regulatory behaviors [29]. Individuals with higher levels of subjective successful aging are expected to perceive their futures as considerably optimistic and open-ended, and vice versa for those with lower levels of subjective successful aging.

Building on the theoretical proposition of socioemotional selectivity theory [30], previous research has demonstrated that future time perception accounts for the observed age differences in goal orientations, social preferences, and emotional and coping responses. Perception of time limitation motivates people to focus on the present moment and emotionally meaningful experiences. Thus, people prefer interacting with emotionally close social partners over acquaintances [31, 32]. Moreover, individuals with limited future time perception utilize more emotion-focused coping to manage emotional experiences [28], report lower levels of subjective well-being [33], and was linked to the substantial use of maladaptive conflict management strategies [34]. By contrast, individuals with an open-ended future time perception exhibit a greater use of problem-focused coping [27, 35], and report greater positive emotions and fewer negative emotions [33]. A recent study has suggested that in the face of the COVID-19 pandemic, older people who perceived themselves as younger than their actual age reported lower levels of loneliness and fewer psychiatric symptoms [36]. Given that individuals with a younger subjective age often perceive more opportunities and remaining time in their lives, thus this study predicts that individuals with open-ended time horizons will report fewer negative emotions than those with limited time perception. Therefore, the present study further investigates whether individuals with higher levels of subjective successful aging would be less likely to have limited time perception, thereby resulting in better emotional well-being and greater utilization of problem-focused coping strategies during the pandemic.

### Emotional and coping responses to the pandemic

Negative emotions are often accompanied with uncontrollable and unexpected life events. In the examination of emotional and coping responses to the SARS outbreak, Hong Kong people, regardless of their age, were found to exhibit intense negative emotions, such as anxiety, fear and worry, and utilized more problem-focused strategies than emotion-focused strategies to cope with the outbreak [37]. Similarly, negative reactions to the COVID-19 pandemic, such as elevated distress, loneliness, and depressive and anxiety symptoms have been reported across the globe [38, 39]. Both problem- and emotion-focused coping strategies have also been adopted by individuals worldwide under the COVID-19 pandemic, such as positive/active coping strategies (e.g., active planning), distractive strategies for regulating emotions (e.g., venting), and avoidant coping strategies [40, 41]. Thus, additional effort is required to examine which factors would predict middle-aged and older adults’ emotional and coping responses to the COVID-19 pandemic. The present study aims to fill this knowledge gap through the lens of subjective successful aging and time limitation.

Different from the SARS outbreak, the Hong Kong government has implemented social distancing practices since 28 March 2020 to prohibit social gatherings of over four people to prevent the spread of the COVID-19 virus [42]. Such a requirement significantly reduces older people’s interactions with their family members and close friends. Given that they are the major sources of emotional satisfaction among middle-aged and older adults [43], these preventive measures may induce more intense negative feelings toward life and future, thereby resulting in elevated frustration and loneliness. It is therefore questioned whether individuals with higher levels of subjective successful aging would adapt to these changes better than those with lower levels of subjective successful aging.

The present study formulated four hypotheses by integrating the models and research on successful aging and lifespan development:

Hypothesis 1: Individuals with higher levels of subjective successful aging will be less likely to report time limitation during the pandemic than those with lower levels of subjective successful aging.

Hypothesis 2: Individuals with higher levels of subjective successful aging will experience more positive and fewer negative emotions than those with lower levels of subjective successful aging.

Hypothesis 3: Individuals with higher levels of subjective successful aging will use more problem-focused and fewer emotion-focused coping strategies than those with lower levels of subjective successful aging.

Hypothesis 4: Perceived time limitation will mediate the longitudinal effects of subjective successful aging on emotional and coping responses. In particular, individuals with higher levels of subjective successful aging are expected to hold a more open-ended time perception during the pandemic than those with lower levels of subjective successful aging, which is subsequently associated with more positive and fewer negative emotions, and more problem-focused and fewer emotion-focused coping.

### The present study

Given that the means of infection and medicine were unknown and unavailable in the early stages of the COVID-19 pandemic, many people, especially middle-aged and older adults who are more vulnerable to greater rates of hospitalization and mortality if infected, were anxious and worried about the threat of infection. To adequately understand individual differences in reactions to this global health calamity, the present study investigates the longitudinal effects of subjective successful aging on the emotional and coping responses to the COVID-19 pandemic. The mediating role of perceived time limitation caused by the pandemic will also be examined. The findings of this study will contribute to the literature on successful aging by unveiling an underlying mechanism between subjective successful aging and emotional and coping responses to the pandemic.

## Methods

### Participants and procedures

A questionnaire was developed to measure middle-aged and older Hong Kong Chinese adults’ responses to COVID-19 pandemic. The participants were recruited from the longitudinal database on Adult Development and Aging. The data of the baseline survey were collected between November 2018 and December 2019. Participants joining the baseline survey were recruited through sending invitation to non-governmental organizations (NGOs) and tertiary institutions serving middle-aged and older adults, advertisement at social media platforms, and convenience sampling. Interested parties were interviewed by trained research assistants in either the Psychology Laboratories of the affiliated university or the community centers of the NGOs. A total of 311 participants were successfully contacted and consented to take part in this COVID-19 questionnaire, thus the response rate was 62.1%. Their mean age was 64.58 (*SD* = 10.14; Range = 45–90 years), 73% were female, and 25.4% were employed during the pandemic. The majority of the respondents (61.1%) had completed secondary school or an associate degree. The participants who joined the COVID-19 study did not differ from those who did not join in terms of their age [*t* (499) = 1.95, *p* = .052], sex (*χ*^2^ (1) = .68, *p* = .409), and level of subjective successful aging [*t* (496) = .65, *p* = .517].

Ethical approval for human research was first obtained from the affiliated university. Except for subjective successful aging (which was measured in the baseline survey of the Adult Development and Aging Project), the variables related to the COVID-19 pandemic were collected between March 29 and April 24, 2020. The commencement date of this study was four days after the HKSAR government’s implementation of border closure to non-residents and compulsory quarantine order for returning residents [44]. The participants who were contacted via instant message or email completed an online survey via the Qualtrics platform, while those who were contacted by phone completed a telephone survey which was conducted by trained research assistants. Informed consent from each participant was sought prior to participation. It took approximately 10 min to complete the questionnaire. Participation in this study was voluntary and no financial incentive was provided.

### Measures

Emotional and coping responses and perceived time limitation during the pandemic were measured in the COVID-19 survey [Supplementary file], whereas subjective successful aging was measured in the baseline survey of the Adult Development and Aging Project in 2019 [45].

#### Emotional responses to the pandemic

With reference to previous studies assessing emotional responses to negative events [37, 46], the participants were asked to rate the extent of their emotional reactions to the COVID-19 pandemic in Hong Kong, including four negative emotions (afraid, anxious, nervous, and upset) and four positive emotions (happy, calm, active, and attentive). In addition, two items were included to measure their feelings in response to reduced social contact owing to social distancing measures: “I felt frustrated because of the reduced social contact” and “I felt lonely because of the reduced social contact.” The 10 emotion items were rated using a five-point scale, ranging from 1 = *not at all* to 5 = *very much*. The Cronbach’s alphas (α) of the negative and positive emotions were .90 and .75, respectively, whereas the alpha of the negative reactions to reduced social contact was .91.

#### Coping strategies

The measurement assessing the participants’ means to cope with the COVID-19 pandemic was adapted from the previous measures of problem-focused and emotion-focused coping strategies used in the SARS outbreak [37], which were developed based on the Brief COPE scale [47]. In particular, four items measured problem-focused coping (active coping, and instrumental support from family members, friends, and mass media) and six items measured emotion-focused coping (self-distraction, behavioral disengagement, positive reframing, use of emotional support, venting toward family members and on social media). The participants indicated how often they used each strategy to cope with the COVID-19 pandemic using a five-point scale, ranging from 1 = *none* to 5 = *always.* Higher scores represent a greater use of the strategies. The Cronbach’s alphas of the problem- and emotion-focused coping strategies were .65 and .66, respectively.

#### Perceived time limitation

Given the time constraint of the telephone survey, only one item was selected and adapted from the Future Time Perspective Scale [48, 49] to measure the participants’ perceived time limitation in response to the pandemic. Specifically, “The COVID-19 pandemic makes me feel that time is running out” was rated using a five-point Likert scale, ranging from 1 = *strongly disagree* to 5 = *strongly agree.* Higher scores represent a stronger sense of limited time remaining in life.

#### Subjective successful aging

The participants’ self-appraisal towards their own aging process were measured by a single statement commonly used in the literature [17, 19, 20]. Specifically, participants were asked to indicate their agreement to the statement “I am aging successfully” on a 7-point Likert scale (1 = *strongly disagree* and 7 = *strongly agree*), with higher scores indicating higher levels of subjective successful aging.

#### Covariates

The participants’ perceived severity of the pandemic and level of reduced social contact due to the social distancing measures were also assessed in the COVID-19 questionnaire. Both questions were rated on a 5-point rating scale (1 = *strongly disagree* and 5 = *strongly agree*), with higher scores representing a greater level of the respective construct. The participants’ demographic variables including age, sex (1 = male and 2 = female), education level (1 = primary school education or below, 2 = secondary school education, and 3 = Bachelor’s degree or above), and depression were extracted from the baseline survey of the Adult Development and Aging Project, whereas their current work status (0 = not employed and 1 = employed) was measured in the COVID-19 questionnaire. Previous studies have indicated that depressed individuals were more likely to hold a fatalistic and pessimistic view towards their future [50–52]. Thereby, depression was also controlled in the analyses as a covariate. The validated Chinese version of the Centre for Epidemiological Studies Depression Scale (CESD-10) [53, 54] was measured in the baseline survey to assess the participants’ dispositional level of depression. As the distribution of CESD-10 scores was highly skewed (skewness = 1.07), a dichotomous variable (0 = *non-depressive* and 1 = *depressive group*) was computed using 9/10 as cutoff points [9, 53] and used in the following analyses.

## Results

### Descriptive statistics

Table 1 presents the means and correlations of the demographic and major variables. Subjective successful aging was significantly correlated with negative and positive emotions (*r* = −.31 and *r* = .40, respectively), negative reactions to reduced social contact (*r* = −.25), problem-focused coping (*r =* .24), emotion-focused coping (*r =* .12), and perceived time limitation (*r* = −.18), *ps* < .05. Moreover, perceived time limitation was significantly correlated with fewer positive emotions and more emotion-focused coping (*r* = −.20 and *r* = .23, respectively*, ps* < .05). In addition, over 62 and 77% of the participants agreed or strongly agreed that the COVID-19 pandemic was severe and reduced their levels of social contact, respectively, indicating substantial impacts of the pandemic on the participants.

**Table 1:** Descriptive statistics and correlations of major variables (*N* = 311)

		Mean (*SD)*/%	1.	2.	3.	4.	5.	6.	7.	8.	9.	10.	11.	12.	13.
1.	Age	64.58 (10.14)													
2.	Sex (Female)	73%	.05												
3.	Education	1.94 (.62)	−.40^**^	−.34^**^											
4.	Work status (Employed)	25.40%	−.51^**^	−.04	.25^***^										
5.	Depression (Depressive group)	32.80%	−.13^*^	.13^*^	−12^*^	.02									
6.	Perceived severity	3.69 (.95)	.03	.08	−.16^**^	−.04	.10								
7.	Level of reduced social contact	4.04 (1.02)	−.10	.07	−.01	.05	.06	.22^***^							
8.	Perceived time limitation	3.32 (1.13)	−.02	.14^*^	−.10	−.01	.14^*^	.19^**^	.09						
9.	Subjective successful aging	5.03 (1.66)	.14^*^	−.07	.11	−.17^**^	−.52^***^	−.09	−.03	−.18^**^					
10.	Negative emotions	2.81 (.03)	.05	.20^***^	−.27^**^	−.10	.31^***^	.32^***^	.19^**^	.35^***^	−.31^***^				
11.	Positive emotions	2.81 (.85)	.17^**^	.08	.01	−.13^*^	−.25^**^	−.00	−.03	−.20^***^	.40^***^	−.28^***^			
12.	Negative reactions to reduced social contact	2.94 (1.14)	.03	.12^*^	−.19^**^	−.07	.25^***^	.30^***^	.25^***^	.26^***^	−.25^***^	.58^***^	−.23^***^		
13.	Problem-focused coping	3.41 (.72)	.03	.12^*^	.03	.02	−.20^***^	.18^**^	.14^*^	.01	.24^***^	.05	.25^***^	.07	
14.	Emotion-focused coping	2.67 (.73)	.09	.15^**^	−.07	−.06	−.05	.21^***^	.11	.23^***^	.12^*^	.32^***^	.18^**^	.25^***^	.36^***^

*Note*. Sex was coded as 1 = male and 2 = female. Education was coded as 1 = primary school education or below, 2 = secondary school education, and 3 = Bachelor’s degree or above. Work status was coded as 0 = not employed and 1 = employed during the pandemic. Depression was coded as 0 = non-depressive group and 1 = depressive group

*p < .05; **p < .01; ***p < .001

### Direct and indirect effects of subjective successful aging on emotional and coping responses

**Fig. 1:**
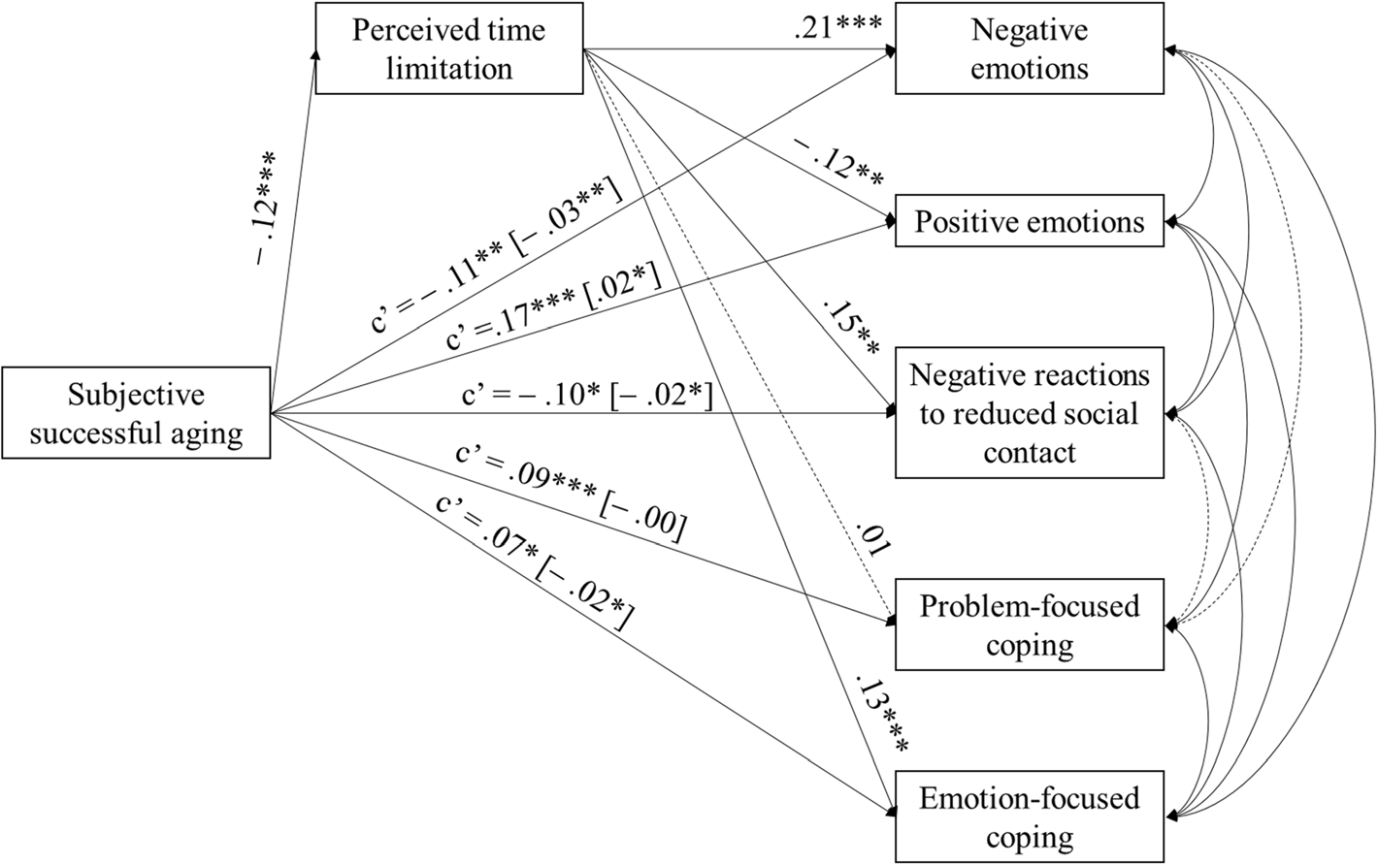
The mediation model on the relationships between subjective successful aging and five outcome variables through perceived time limitation. *Note.* Numbers in the figure indicate unstandardized regression coefficients. c’ denotes the direct effect of subjective successful aging on each outcome variable. The numbers in the parentheses represent the indirect effect of subjective successful aging on the outcome variable through perceived time limitation. The solid lines denote significant associations between variables at *p* < .05, whereas dashed lines represent non-significant associations. Age, sex, education level, work status, depression, perceived severity, and level of reduced social contact were statistically controlled as covariates in the mediation analysis. Goodness of fit of this mediation model: *χ*^2^ = 14.58 (*df* = 7, *p* = .04), CFI = .98, RMSEA = .06, SRMR = .03. *p < .05; **p < .01; ***p < .001

To test the four hypotheses, a mediation analysis was performed using the R package lavaan [55] with subjective successful aging as the independent variable, perceived time limitation as the mediator, and emotional and coping responses as the five dependent variables. Age, sex, education level, work status, depression, perceived severity of the pandemic, and level of reduced social contact were controlled as covariates because they were significantly correlated with subjective successful aging, perceived time limitation, or emotional and coping responses. Figure 1 presents the results of the mediation model and Table 2 reports the total, direct and indirect effects.

**Table 2:** Total, Direct and Indirect Effects of Subjective Successful Aging on Emotional and Coping Responses through Perceived Time Limitation

	Negative Emotions		Positive Emotions		Negative Reactions to Reduced Social Contact		Problem-focused Coping		Emotion-focused Coping	
	*B*	*SE*	*B*	*SE*	*B*	*SE*	*B*	*SE*	*B*	*SE*
Total effect	−.13***	.04	.19***	.03	−.12**	.04	.09**	.03	.05*	.03
Direct effect	−.11**	.04	.17***	.03	−.10*	.04	.09**	.03	.07*	.03
Indirect effect through perceived time limitation [95%CI]	−.03**	.01	.02*	.01	−.02*	.01	−.00	.00	−.02*	.01
	[−.05, −.01]		[.00, .03]		[−.04, −.00]		[−.01, .01]		[−.03, −.00]	

*Note*. In the mediation model, subjective successful aging and perceived time limitation were inputted as the independent variable and mediator, respectively. Age, sex, education level, work status, depression, perceived severity, and level of reduced social contact were statistically controlled as covariates in mediation analysis

*p < .05; **p < .01; ***p < .001

The results of the mediation analysis showed that the participants with higher levels of subjective successful aging were less likely to report time limitation during the pandemic than those with lower levels of subjective successful aging (*B* = −.12, *SE* = .04, *p* = .001). Therefore, H1 is supported. In terms of the direct effects proposed in H2 and H3, subjective successful aging was found to be predictive of all five dependent variables, including negative emotions (*B* = −.11, *SE* = .04, *p* < .001), positive emotions (*B* = .17, *SE* = .03, *p* < .001), negative reactions to reduced social contact (*B* = −.10, *SE* = .04, *p =* .016), problem-focused coping (*B* = .09, *SE* = .03, *p =* .001), and emotion-focused coping (*B* = .07, *SE* = .03, *p* = .015), even after controlling for the seven covariates. However, the direct effect of subjective successful aging on emotion-focused coping was positive, which was contradictory to the hypothesized pattern. Consequently, H2 is supported, while H3 is partially supported.

The significant indirect effects of subjective successful aging through perceived time limitation were found on positive emotions (*B* = .02, *SE* = .01, *p* = .026), negative emotions (*B* = −.03, *SE* = .01, *p* = .007), negative reactions to reduced social contact (*B* = −.02, *SE* = .01, *p* = .028), and emotion-focused coping (*B* = −.02, *SE* = .01, *p* = .013). However, such effects were not observed on problem-focused coping. Consequently, H4 is partially supported.

## Discussion

The outbreak of COVID-19 has produced severe threats to public health and has caused substantial economic and social stresses globally. The pandemic has also created continuous interruptions and changes to daily life and social relationships. Middle-aged and older adults are particularly at risk because of higher rates of infection and severe symptoms [56]. This study attempted to advance the current literature on successful aging by investigating the responses of these two vulnerable age groups to this novel disease. The results of this study demonstrated a positive indirect effect of subjective successful aging on affective well-being whereas a negative indirect effect of subjective successful aging on the use of emotion-focused coping through perceived time limitation during the COVID-19 pandemic.

### Effects of subjective successful aging on responses to the pandemic

Previous studies have demonstrated that internalized views of aging increase people’s utilization of preventive health services [57] and adaptive behaviors [23]. Some studies have revealed the negative impacts of the COVID-19 pandemic on well-being such as elevated distress, loneliness, depressive and anxiety symptoms during the COVID-19 pandemic [38, 39], yet little is known about the protective factors that influence the way individuals respond to this crisis. By investigating on its influence on emotional and coping responses in the face of the pandemic, the present study advances the current literature on successful aging and reveals that holding positive self-appraisal towards the aging process enables individuals to actively utilize adaptive strategies (such as active planning and use of instrumental support) to deal with the changes and interruptions caused by the pandemic and the implementation of social distancing practices in society. Despite that the pandemic unavoidably brings certain negative impacts on individuals [38, 39], our findings suggest that having initial positive views on one’s own aging process may serve as a protective factor to mitigate some of these negative impacts, such as reduced negative emotions caused by the pandemic and less social contact. However, contrary to our prediction in H3, higher levels of successful aging were associated with greater use of emotion-focused coping strategies. One possible explanation for this phenomenon may be due to that the use of emotion-focused strategies is sometimes beneficial when the confronting stressors are deemed uncontrollable [58, 59]. Given the pandemic is to some extent uncontrollable in nature, successful agers, who might be linked to the heightened general tendency to regulate emotions [24], therefore might make use of both problem- and emotion-focused coping strategies to cope with the practical and emotional burdens brought by the pandemic. By contrast, individuals with lower levels of subjective successful aging showed intense negative emotions and did not actively cope with the COVID-19 outbreak. Thus, the findings of this study further demonstrate the beneficial effects of subjective successful aging on well-being even in the face of the COVID-19 pandemic.

### Mediating effects of perception of time limitation

The socioemotional selectivity theory [30] stresses that when people perceive that their future is running out of time, their goal orientations will shift from knowledge-related goals to emotional goals. Previous studies have demonstrated that perceptions of future time, either self-rated or experimentally manipulated, account for the age-related differences in social preferences [32], emotional experiences [33], and use of coping strategies [35, 60]. Similar to other past life-threatening crises, such as the SARS outbreak in 2003 and the September 11 attacks, the COVID-19 pandemic is accompanied with a wide range of uncertainties for the future, thereby resulting in a heightened sense of time limitation. The present findings further showed that greater perceived time limitation was prevalent among the participants with lower levels of subjective successful aging than those with higher levels of subjective successful aging. As predicted, such a heightened sense of time limitation was associated with more negative and fewer positive emotions, and greater utilization of emotion-focused coping strategies. As a result, a positive indirect effect of subjective successful aging on affective well-being and a negative indirect effect of subjective successful aging on the use of emotion-focused coping through perceived time limitation during the COVID-19 pandemic were observed among the participants. This finding is also in line with a recent study on the COVID-19 pandemic in which heightened loneliness and psychiatric symptoms were shown among the individuals who perceived themselves older than their actual age [36]. As subjective age is highly correlated with future time perspective [61], where those who view themselves as older than their actual age perceive their futures to be less open-ended, our findings further advance the literature by recognizing the protective effects of holding an open-ended time perception amid a pandemic. In addition, this study contributes to the current literature by unveiling a mechanism underlying the relationships between internalized views of successful aging and responses to the pandemic through perceived time limitation. Consistent with one of the propositions of the socioemotional selectivity theory [30], the perception of future time plays an important role in predicting emotional and behavioral responses, even in the face of life-threatening situations.

From the experiences in the SARS outbreak in 2003 [37], Hong Kong people, compared with people in other countries, generally show a higher tendency to perform preventive measures to reduce the likelihood of COVID-19 infection, such as wearing surgical masks, washing hands regularly, or seeking instrumental support from family members and friends. Contrary to our prediction, the present study did not find any significant main or mediating effects of perceived time limitation on problem-focused coping. Instead, subjective successful aging was found to be positively associated with the use of problem-focused coping during the pandemic. In line with previous findings [26, 27], successful agers hold positive perceptions of their capabilities to manage life challenges and adversities, thus show a greater tendency to proactively deal with the stressors [26] and utilize active planning and adaptive strategies [62].

Practical implications can be drawn from the beneficial effects of cultivating higher levels of subjective successful aging shown in the present study. As subjective successful aging involves self-evaluations of one’s own aging process in the face of inevitable functional decline, individuals with higher levels of subjective successful aging are those with higher perceived capacities in managing age-related losses. Therefore, social activities and intervention programs targeting older adults should focus on cultivating their perceived capabilities to deal with life challenges. For example, volunteering activities and group-based interventions can cultivate one’s sense of control [63], resilience [64], or empowerment [65]. These values can foster a greater sense of subjective successful aging and promote adaptive coping during adverse events such as a pandemic.

### Limitations and future directions

The current study integrates the data collected before the COVID-19 pandemic (i.e., subjective successful aging) and during its peak (i.e., perceived time limitation and emotional and coping responses), and reveals a mechanism underlying the relationships between subjective successful aging and responses to the coronavirus and social distancing measures in Hong Kong. However, several limitations should be considered when interpreting the previously discussed findings. First, despite the fact that the single-item measure for subjective successful aging was commonly used in previous studies [17, 19], and was found positively correlated with self-rated health (*r* = .35, *p* < .001) and perceived mastery (*r* = .57, *p* < .001) in the entire sample of the baseline survey of the Adult Development and Aging project (*N* = 501), its construct validity may not be well demonstrated in the present study. Future studies may consider assessing subjective successful aging with a more comprehensive assessment. Similarly, with the consideration of keeping each telephone interview short, perceived time limitation was only measured by a single item. Thus, the internal consistency could not be shown in the present study. Even though this measure negatively correlated with the participants’ perceived life expectancy (which was measured in the Adult Development and Aging Project), future research is recommended to employ the focus on limitations subscale (3-item) of the Future Time Perspective Scale [66] to obtain a comprehensive measure of this construct. Second, the current study was conducted involving a sample of Hong Kong Chinese adults, whose emotional and coping responses may not be generalized to other countries because the total infected cases in Hong Kong remained low (1035 confirmed cases as of 24 April 2020). Compared with other countries with over 100,000 infected cases and stringent lock-down measures, such as the United States, Italy and Spain, the threat perceived by the Hong Kong people may be relatively lower, thereby possibly facilitating more “adaptive” responses and behaviors. Third, this study only involved one assessment of emotional and coping responses to the pandemic. In view of the rapidly changing development of the COVID-19, future studies should consider adopting a daily diary design to understand the individual changes in emotional feelings and coping strategies over time. Fourth, approximately 3% of the participants in the current sample reported that their family members and friends were infected with COVID-19. It remains an open question how people’s time perceptions and emotional and coping responses would be affected if their emotionally close social partners have been infected. Future studies should explore whether the mediating effects of perceived time limitation found in the present study will also be observed among COVID-19 patients or those with infected family members. Lastly, even though we had utilized longitudinal data to examine the mediating relationships among subjective successful aging, perceived time limitation, and responses to the pandemic, the causality between these constructs awaits further investigation. It is possible that one’s overall future time perspective influences his/her views towards successful aging and consequently affects his/her reactions to the COVID-19 outbreak. Future studies can utilize an experimental design to clarify their associations.

## Conclusions

To conclude, this study disclosed that perceiving oneself as successfully aging facilitates adaptive coping and affective well-being among middle-aged and older adults even in the face of the COVID-19 pandemic. This research also revealed the mediating role of perceived time limitation in the associations between subjective successful aging and emotional responses and emotion-focused coping. The findings of this study indicated that building positive views of old age and perceived capacities to achieve successful aging, particularly through fostering their optimistic views of the future, is beneficial to buffer older adults from suffering from stressful events, such as the COVID-19 pandemic.

## Supplementary Information

**Additional file 1.**

## Data Availability

The datasets used and/or analyzed during the study are not publicly available due to the depository of the longitudinal database on Adult Development and Aging is still in progress but the data of the COVID-19 study are available from the corresponding author on reasonable request.

## Abbreviations

COVID-19: Coronavirus Disease 2019
HK: Hong Kong
HKSAR: Hong Kong Special Administrative Region
SARS: Severe Acute Respiratory Syndrome
