# Supplementary material for: Effects of subjective successful aging on emotional and coping responses to the COVID-19 pandemic
Source: BMC Geriatr. 2021 Feb 17;21:128. doi: 10.1186/s12877-021-02076-2 (PMC7887561; doi:10.1186/s12877-021-02076-2)
Supplement: Supplementary file 1 — Additional file 1. [file 12877_2021_2076_MOESM1_ESM.docx]

Supplementary File 1

Survey on Hong Kong People’s Responses to COVID-19 Pandemic

Matching ID: _________

Current Work Status: Not employed / Employed

Please indicate to what extent do you agree with the following statement on a scale ranging from 1 (strongly disagree) to 5 (strongly agree).

|  | Strongly disagree | Disagree | Neutral | Agree | Strongly agree |
| --- | --- | --- | --- | --- | --- |
| The COVID-19 pandemic makes me feel that time is running out | 1 | 2 | 3 | 4 | 5 |
| I think the COVID-19 pandemic is severe in Hong Kong | 1 | 2 | 3 | 4 | 5 |
| I reduce social contact with my family members or friends (e.g., reducing the frequency of meeting family members who do not live with me) | 1 | 2 | 3 | 4 | 5 |

Please indicate how often do you experience the following emotions in response to the COVID-19 pandemic on a scale ranging from 1 (not at all) to 5 (very much).

|  | Not at all | A little | Moderately | Quite a bit | Very much |
| --- | --- | --- | --- | --- | --- |
| Upset | 1 | 2 | 3 | 4 | 5 |
| Afraid | 1 | 2 | 3 | 4 | 5 |
| Active | 1 | 2 | 3 | 4 | 5 |
| Attentive | 1 | 2 | 3 | 4 | 5 |
| Nervous | 1 | 2 | 3 | 4 | 5 |
| Anxious | 1 | 2 | 3 | 4 | 5 |
| Calm | 1 | 2 | 3 | 4 | 5 |
| Happy | 1 | 2 | 3 | 4 | 5 |
| I felt frustrated because of the reduced social contact | 1 | 2 | 3 | 4 | 5 |
| I felt lonely because of the reduced social contact | 1 | 2 | 3 | 4 | 5 |

Please indicate how often do you adopt the following strategies to cope with the pandemic on a scale ranging from 1 (none) to 5 (always).

|  | None | Rarely | Sometimes | Very often | Always |
| --- | --- | --- | --- | --- | --- |
| I get emotional support from others or in my religion or spiritual beliefs | 1 | 2 | 3 | 4 | 5 |
| I take action to try to reduce the risk of infection (e.g., wearing a mask, washing hands frequently) | 1 | 2 | 3 | 4 | 5 |
| I get help and advice from my family members | 1 | 2 | 3 | 4 | 5 |
| I get help and advice from my friends | 1 | 2 | 3 | 4 | 5 |
| I get help and advice from mass media | 1 | 2 | 3 | 4 | 5 |
| I reduce exposure to social media (e.g., Facebook, twitter) to avoid receiving too much information about the COVID-19 pandemic | 1 | 2 | 3 | 4 | 5 |
| I look for something good in what is happening (e.g., the medicine for the COVID-19 pandemic is being invented) | 1 | 2 | 3 | 4 | 5 |
| I do something to think about the COVID-19 pandemic less (e.g., watching movies at home, reading, watching TV, or sleeping) | 1 | 2 | 3 | 4 | 5 |
| I express my negative feelings to my family members or friends | 1 | 2 | 3 | 4 | 5 |
| I express my negative feelings on social media | 1 | 2 | 3 | 4 | 5 |
